# Pollen-mediated gene flow from transgenic cotton under greenhouse conditions is dependent on different pollinators

**DOI:** 10.1038/srep15917

**Published:** 2015-11-03

**Authors:** Shuo Yan, Jialin Zhu, Weilong Zhu, Zhen Li, Anthony M. Shelton, Junyu Luo, Jinjie Cui, Qingwen Zhang, Xiaoxia Liu

**Affiliations:** 1 Department of Entomology, China Agricultural University, Beijing, 100193, P.R. China; 2 National Agricultural Technology Extension and Service Center, Beijing, 100125, P.R. China; 3 Beijing Entry-Exit Inspection and Quarantine Bureau, Beijing, 100026, P.R. China; 4 Department of Entomology, Cornell University/New York State Agricultural Experiment Station, Geneva, NY, 14456, USA; 5 State Key Laboratory of Cotton Biology, Institute of Cotton Research, Chinese Academy of Agricultural Sciences, Anyang, Henan, 455000, P.R. China

## Abstract

With the large-scale release of genetically modified (GM) crops, there are ecological concerns on transgene movement from GM crops to non-GM counterparts and wild relatives. In this research, we conducted greenhouse experiments to measure pollen-mediated gene flow (PGF) in the absence and presence of pollinators (*Bombus ignitus*, *Apis mellifera* and *Pieris rapae*) in one GM cotton (resistant to the insect *Helicoverpa armigera* and the herbicide glyphosate) and two non-GM lines (Shiyuan321 and Hai7124) during 2012 and 2013. Our results revealed that: (1) PGF varied depending on the pollinator species, and was highest with *B. ignitus* (10.83%) and lowest with *P. rapae* (2.71%); (2) PGF with *B. ignitus* depended on the distance between GM and non-GM cottons; (3) total PGF to Shiyuan321 (8.61%) was higher than to Hai7124 (4.10%). To confirm gene flow, we tested hybrids carrying transgenes for their resistance to glyphosate and *H. armigera*, and most hybrids showed strong resistance to the herbicide and insect. Our research confirmed that PGF depended on pollinator species, distance between plants and the receptor plant.

Genetic engineering has made it possible to transfer novel genes into plants, thus resulting in the production of genetically modified (GM) varieties conferring resistance to insects^1^, diseases^2^, herbicides^3^ and environmental stress^4^,^5^. The steady growth in global plantings of GM crops attests to their advantages for producers^6^. Despite these advantages, the production of GM crops raises concerns about their impacts on the environment^1^,^5^,^9^,^10^,^11^.

Critical environmental questions include whether introduced genes from GM crops will move to other plants, what would be the consequences of such movement, and whether the movement can be limited^4^,^12^. Pollen-mediated gene flow (PGF), defined as the dispersal of crop genes via pollen, has became a primary focus of risk assessment because of the potential for GM pollen to move to non-GM plants^3^,^13^,^14^,^15^. PGF is affected by many factors, including whether the crop is wind- or insect-pollinated, the distance of pollen dispersal, and the probability of fertilization^13^,^16^,^17^,^18^,^19^,^20^. Some studies have shown that PGF usually declines exponentially with increasing distance between the GM pollen donor and non-GM pollen recipient^11^,^15^,^20^,^21^,^22^, and the presence of pollinators, including the honeybee, *Apis mellifera*, which increased PGF^18^,^19^,^23^. Thus, the absence of efficient pollinators and long distance are important for attenuating the environmental risk. What has been less studied is the impact of pollinator species on PGF.

Cotton (*Gossypium hirsutum* L.) is primarily grown as an irrigated crop during summer in three major ecological regions of China: the Changjiang River Region, the Yellow River Region and the North-western Region. Cotton production is constrained by lepidopteran pests, especially *Helicoverpa armigera*^18^. Transgenic cotton, producing insecticidal proteins from *Bacillus thuringiensis* (Bt) has been used since 1997 in China to effectively suppress lepidopteran pests^24^,^25^. Cotton is primarily a self-pollinating plant, but a small amount of cross-pollination can occur in the presence of pollinators, and the large and sticky cotton pollen makes pollinators potentially important in cross-pollination^18^,^19^,^26^. Some studies have shown that PGF in cotton varied greatly depending on multiple factors, including location, environmental condition, the time period (different years) and the method used to measure the crossing rate^16^,^19^,^20^,^27^,^28^. However, the PGF due to different pollinator species has been less studied. Some researches confirmed that *Bombus ignites* and *Apis mellifera* are effective pollinators in the field and greenhouse^29^,^30^, and they are both used widely because of their commercial availability. Although *Pieris rapae* is not commercialized as a pollinator, its ability to pollinate plants has been reported^31^. In China where farmers primarily have small units of land on which they grow multiple crops, it is common to have crucifer crops, the host of *P. rapae*, near cotton. Thus, *P. rapae* serves as an example of potential pollinators for cotton and should be considered in a risk assessment for the spread of cotton transgenes.

In the present study, we used a GM cotton expressing Bt for insect resistance and resistance to a broad-spectrum herbicide (glyphosate) as the pollen donor. To measure PGF in GM cotton using the combination of molecular techniques and biological assay, we conducted greenhouse experiments with pollinators, including *B. ignites*, *A. mellifera* and *P. rapae*, and without pollinators, using one GM cotton and two non-GM counterparts. Our goals in the study were to: (1) measure PGF with different pollinators, (2) determine the influence of distance between GM pollen donor plants and non-GM pollen receptor plants on PGF, (3) analyze the impact of different non-GM cotton receptor plants on PGF, (4) confirm the insect and glyphosate resistance of hybrids.

## Results

### Greenhouse observation

The agronomic behavior of GM and non-GM cottons was similar to a standard cultivar in the field. Good cotton growth and flower production were achieved in three greenhouses during two years of this study. Time of flowering was synchronous between the GM and non-GM cotton throughout the flowering period, thus ensuring ample opportunity for pollen transfer. Insect proof nets prevented pollinators moving into the control groups. Wind velocities in the greenhouses were very low (<0.2 m/s) (Figure S1), minimizing any influences of wind on PGF.

### Pollen-mediated gene flow in the absence and presence of pollinators

A total of 10,080 seeds (60 seeds/sample ×2 pollen receptors ×14 distances ×3 pollinators ×2 yr) were sampled to assess PGF by DNA analyses. PCR amplification products were consistently detected and clearly visible in the GM pollen donor and progeny from cross-pollination between GM and non-GM crops (Figure S2).

Hybridization was detected during the 2 yr of the study, and varied by pollinator species and pollen receptor. More specifically, the average PGF of all sampled distances and both pollen receptors during the two yr were 10.83%, 5.52% and 2.71% for the pollinators *B. ignitus*, *A. mellifera* and *P. rapae*, respectively (*F*_2,285_ = 27.971, *P* < 0.001). For both the two pollen receptors during the 2 yr of the study, the average PGF of all sampled distances and the three pollinators to the pollen receptor of Shiyuan321 (8.61%) was higher than that of Hai7124 (4.10%) (*t* = 4.768, df = 286, *P* < 0.001).

**Figure 1:**
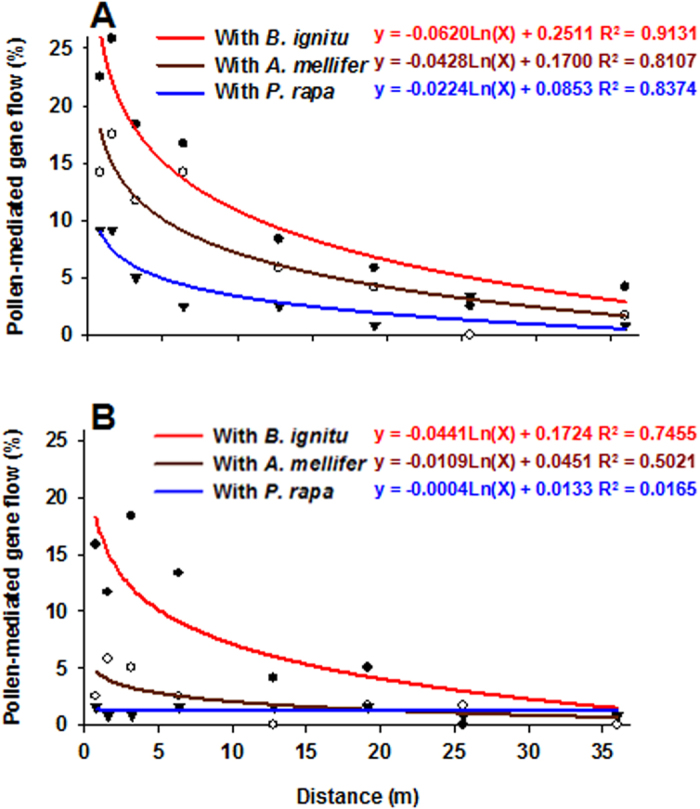
Regression analysis for the pollen-mediated gene flow from GM cotton to Shiyuan321 (A) and Hai7124 (B) as a function of the distance. The correlation coefficient (R^2^) between PGF and distance was obtained using SigmaPlot 10.0 software. Regression analysis was based on the data of the two year study.

Generally, PGF declined with increasing distance between GM and non-GM cottons (Table 1, Fig. 1), especially in the treatment with *B. ignitus* (2012 Shiyuan321: *F*_7,16_ = 10.019, *P* < 0.001; Hai7124: *F*_7,16_ = 7.048, *P* = 0.001; 2013 Shiyuan321: *F*_7,16_ = 3.879, *P* = 0.012 ; Hai7124: *F*_7,16_ = 3.194, *P* = 0.026). PGF to Shiyuan321 with *B. ignitus* ranged from 30.00% at 1.6 m to 0.00% at 36 m in 2012 and from 21.67% at 1.6 m to 1.67% at 25.6 m in 2013. PGF to Hai7124 with *B. ignitus* ranged from 21.67% at 3.2 m to 0.00% at 36 m in 2012 and from 15.00% at 3.2 m to 0.00% at 25.6 m in 2013. In the treatment with *A. mellifera*, the PGF also significant decreased with increasing distance between GM and non-GM cottons in the pollen receptors plant Shiyuan321(2012: *F*_7,16_ = 8.258, *P* < 0.001; 2013: *F*_7,16_ = 3.109, *P* = 0.029), although there were not significant differences at all sampled distances in the pollen receptor cotton Hai7124 (2012: *F*_7,16_ = 0.825, *P* = 0.581; 2013: *F*_7,16_ = 0.762, *P* = 0.626). For the pollen receptor Shiyuan321, PGFs with *P. rapae* showed significant differences in 2012 (*F*_7,16_ = 3.918, *P* = 0.011), but not in 2013 (*F*_7,16_ =  = 1.582, *P* = 0.211). For the receptor Hai 7124, PGFs with *P. rapae* in the both years were not related to distance (2012, *F*_7,16_ = 0.365, *P* = 0.910; 2013, *F*_7,16_ = 0.429, *P* = 0.870). Hybrids were not detected in the control group of three greenhouses during the 2 yr of the study.

Hybrids expressing *Cry I Ac* were tested for Bt protein detection (Figure S3). Overall, there was low rate of false positives in Bt protein detection. During the 2 yr of the study, the false positives were 3.23% and 2.54% to the pollen receptors of Shiyuan321 and Hai7124, respectively.

### Resistance test of hybrid

#### Insect resistance

**Figure 2:**
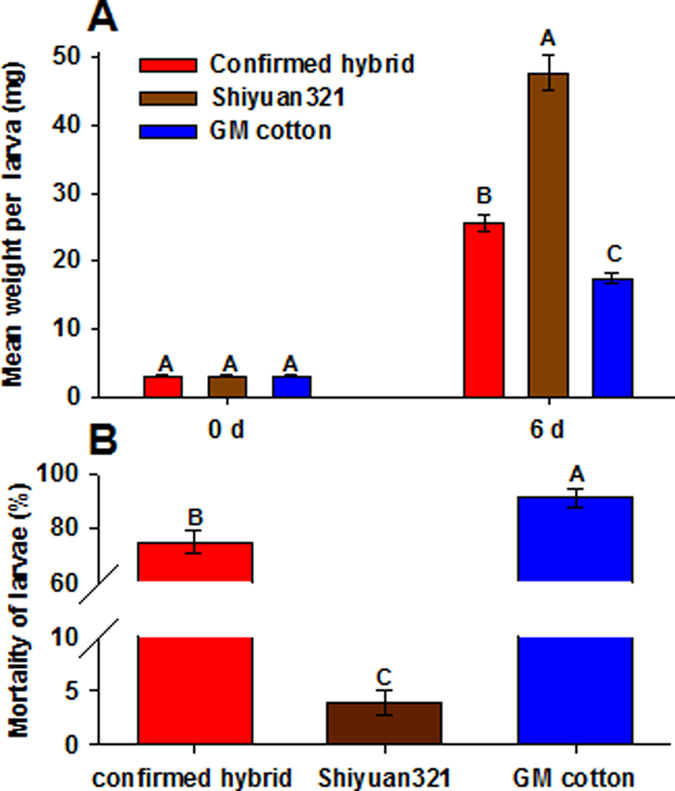
Insect resistance detection of confirmed hybrid, Shiyuan321 and GM cotton. (**A**) Weight of *H. armigera* larvae fed on diet containing confirmed hybrid, Shiyuan321 and GM cotton. (**B**) Mortality effects of confirmed hybrid, Shiyuan321 and GM cotton on *H. armigera* larvae. Means ± SE within the same day after treatment and different diet followed by different letters are significantly different (Tukey’s multiple comparison test, *P* < 0.05).

The confirmed hybrids (progeny expressing Bt protein) were tested for insect resistance. The hybrid and GM cotton leaves significantly decreased the larval weight compared to non-GM cotton (Fig. 2A, *F*_2,6_ = 81.161, *P* < 0.001). The mortality of larvae fed on hybrids or GM cotton at the sixth day was higher than the control (*F*_2,6_ = 211.240, *P* < 0.001), although the mortality of insects fed on confirmed hybrids reached 75.00 ± 4.19%, and this was slightly lower than 91.67 ± 3.47% of the GM cotton (Fig. 2B).

#### Herbicide tolerance

Confirmed hybrids were screened for resistance to glyphosate. As shown in Table 2, non-GM cotton (Shiyuan321) grew slowly and was significantly shorter than GM cotton and the confirmed hybrids after glyphosate application, and none survived 42 d after glyphosate application. Although the height of confirmed hybrids was significantly shorter than that of GM cotton during 7 to 14 d, there was no significant difference with GM cotton until 28 d after glyphosate application. Most of confirmed hybrids showed resistance to glyphosate, and the survival rate reached 82.78% at 42 d after glyphosate application.

## Discussion

The risk of gene flow can occur within species, between species, between the higher plants and other organisms, but the most probable gene flow would occur between GM crops and their non-GM lines, or related wild species^32^. Molecular analysis offers a simple, effective and accurate method for measuring PGF^3^,^15^,^23^,^33^,^34^,^35^. In the present study, we used a combination of molecular methods and biological assays to measure PGF within species, the GM cotton with both *Cry I Ac* and *CP4 EPSPS* genes and non-GM cotton. We collected a total of 10,080 cotton seeds during the 2-yr study and then grew them to the 3-leaf stage to study PGF in the greenhouse with pollinators. Past studies on PGF were mainly conducted in fields^11^,^33^,^34^,^36^. Although these studies provided general patterns on PGF, it was difficult to evaluate the effects of an individual factor. Our study was conducted in greenhouses, which provided a relatively closed environment to avoid the effects of wind and other pollinators on PGF. We observed that PGF did occur at a higher frequency during our 2-yr study, compared to that in fields^4^,^12^,^16^,^37^. More importantly, our study was able to parse out the influences of various factors.

In our present study, we found that PGF varied with different pollinators, with *B. ignitus* and *A. mellifera* having higher PGF rates than *P. rapae*. Although the studies were conducted in the three greenhouses, the temperature and other environmental conditions were the same. We attribute the differences of PGF to be caused by different pollinators. *B. ignitus* and *A. mellifera* are widely used as agricultural pollinators^29^,^38^,^39^, especially *B. ignitus*. Bumblebees are effective pollinators because of the amount of pollen they collect, their working time and speed, frequency of visiting flowers and ability to work under a range of temperatures^29^,^38^,^39^,^40^,^41^,^42^. They often remove all available nectar from flowers in a single visit^38^, and deposit more pollen than honeybees^39^. The larger surface area of bumblebees allows more contact with stigmas and more pollen deposition compared to honeybees^39^,^43^,^44^. These merits of bumblebees provide better pollination, reduce geitonogamy, increase pollen flow distance, and thereby increase outcrossing rates^40^,^45^,^46^. On the other hand, honeybees are more sensitive to temperature, show more phototaxis, and need more time to adapt to the greenhouse condition compared to bumblebees^30^,^47^,^48^. Thus, these factors led to the higher PGF in the greenhouse with bumblebees, and potentially the higher environmental risk in the fields. Therefore, we suggested that *B. ignites* should not be used as a supplemental pollinator in GM crops. Although *P. rapae* adults provided pollination services in a botanical garden^31^, it was not an effective pollinator in the cotton field. Thus, it would provide almost no risk in the GM cotton field.

Umbeck *et al.*^16^ measured out-crossing from GM cotton to non-GM cotton in Mississippi using a seed germination assay and PCR analysis, and found that out-crossing went from 5.7% to < 1% within 7 m from the test plot, and <1% out-crossing continued to occur sporadically to a distance of 25 m. Van Deynze *et al.*^19^ found PGF from GM cotton declined exponentially with increasing distance from 7.65% at 0.3 m to  < 1% beyond 9 m even with high pollinator activity by *A. melifera*. In the case of GE plums, the PGF ranged from 0.215% at 132 m from the center of the plot to 0.033-0.017% at longer distances (384–998 m)^15^. These results are similar to data gathered in Fujian province, China, where PGF from GM rice ranged from 0.28% at 0.2m to < 0.01% at 6.2 m^12^. Similar to these and other studies^11^,^17^,^22^,^49^, we found PGF with *B. ignitus* declined with increasing distance. PGF in the presence of pollinators would decrease significantly with increasing distance from GM plants not only because of decreased diffusion of the GM pollen and pollinator movement over the distance, but also because density of non-GM pollen would increase. One the other hand, the pollen grains may gradually sediment under the action of gravity after shedding during the dispersal. Furthermore, as GM pollen travels over longer distances and time, they may suffer slightly losses of competitiveness compared to non-GM pollen. These combined effects would lead to the sharp reduction of PGF with increased distance. Based on our results and the literature cited, it is apparent that increasing isolation distances is an effective method to minimize environmental risk for self-pollinating crops.

Biological confinement strategies (male sterility and gene insertion into chromosomes) might influence gene flow frequency. PGF from GM bentgrass, *Agrostis stolonifera*, to non-GM *A. stolonifera* (91 of 168) was higher than that to *A. gigantea* (13 of 39) as assessed by seedling progeny survival after spraying with glyphosate and PCR analysis^50^. Our experiment also showed that total PGF to the pollen receptor Shiyuan321 was higher than to Hai7124. The flowering time of cotton plant is longer than with bentgrass, and we chose cotton varieties with a similar growth period for our study. Therefore, we eliminated the effects of differences of flowering time on PGF. Differences in PGF of the two receptors were likely due to the genetic background because Shiyuan321 was the parent of the GM cotton and the relationship between the transgenic cotton and Shiyuan321 is closer than that to Hai7124. It is easier to hybridize with lines having a closer genetic relationship. The potential for out-crossing is highly dependent on sexual compatibility and relatedness between the parent species, and the opportunity for natural hybridization between two plants depends on many pre- and post-zygotic factors^51^.

PGF can be determined directly by progeny resistance detection because most of confirmed hybrids showed some resistance to glyphosate and targeted pests. For example, PGF was determined by seedling progeny survival after spraying with glyphosate^4^,^19^,^37^. In the current study, the confirmed hybrids showed strong resistance to both glyphosate and *H. armigera*. However, we think it is more reliable to determine PGF by PCR analysis. The reasons are (1) the resistance of confirmed hybrids was weaker than that of the GM cotton, (2) seedlings expressing resistance genes might not express functional proteins, although the level of false positive was very low in our present research.

In conclusion, our studies quantified the extent of PGF in cotton under greenhouse conditions in the presence of pollinators. Our results indicated that (1) PGF was dependent on pollinator species, and was highest with *B. ignitus* and lowest with *P. rapae*. (2) PGF with *B. ignitus* dropped exponentially with increasing distance. However, the level of PGF with *P. rapae* was very low and not obviously influenced by the distance. (3) Total PGF to the pollen receptor Shiyuan321 was higher than that for Hai7124. (4) Most confirmed hybrids using molecular methods showed strong resistance to glyphosate and *H. armigera* larvae. This study suggests that PGF was dependent on pollinator species, distances, and the receptor and suggests that these factors are important in a risk assessment.

## Methods

### Plant materials

In current study, the inbred line Shiyuan321 and island cotton Hai7124 were selected as the pollen receptors. The GM cotton as the pollen donor were obtained by transferring the insect resistance gene *Cry I Ac* and the glyphosate resistance gene *CP4 EPSPS* from *Agrobacterium tumefaciens* into the embryonic calli of Shiyuan321 via microprojectile bombardment. The promoters (mostly the 35S promoter of cauliflower mosaic virus) in GM cotton enhance the target gene expression in seeds and seedlings, so that even hemizygous progeny resulting from the cross-pollination with a non-GM cultivar can be easily identified by a variety of molecular analyses^15^,^52^,^53^,^54^. The GM and non-GM cottons have similar growth patterns and flower structure^20^. All plants were obtained from the Cotton Research Institute, Chinese Academy of Agricultural Sciences, and were purified by consecutive two-generations of self-crosses, to ensure that each line was homozygous.

### Greenhouse layout and procedures

To evaluate the impacts of pollinators on PGF from a GM cotton to other non-GM cultivars, the trials were carried out in three greenhouses (25–28 °C, L60 m × W8 m × H4 m) in the Shangzhuang Experimental Station of China Agricultural University (40°14′N, 116°19′E), Beijing, China in 2012 and 2013. Each greenhouse contained one type of pollinator (*Bombus ignitus*, *Apis mellifera,* provided by the Bee Research Institute, Chinese Academy of Agricultural Sciences, or *Pieris rapae* reared in our lab). One small colony of honeybees (ca 4000 workers), one colony of bumblebees (ca 80 workers) and 80 butterflies were released in the greenhouses to evaluate gene flow. The appropriate quantity of each pollinator was determined by an expert in pollination, and appropriate for pollination in our greenhouse (60 m × 8 m). It has been reported some Lepidoptera act as pollinators of cotton^55^, and we found that there was high population density of *P. rapae* in cotton fields near the greenhouses in the current study. *B. ignitus* and *A. mellifera*, commonly used as agricultural pollinators, are effective pollinators in fields and greenhouses^29^,^30^,^38^,^39^,^47^. Thus, the three species were selected as pollinators in the present study. Wind speeds were monitored at 6:00, 14:00 and 22:00 using an anemograph (ZRQF-F303, Beijing Detector Instrument Co., Beijing, China) in the greenhouses^20^.

**Figure 3:**
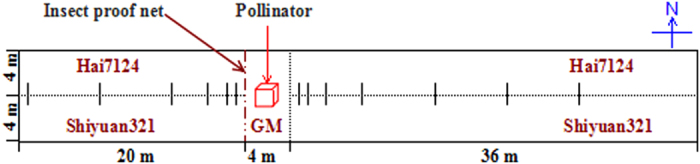
Greenhouse trial design to measure gene flow from GM cotton to conventional lines during 2012 and 2013. The GM cotton was planted in a 4 m × 8 m square in the middle of a 60 m × 8 m square planting of non-GM cotton. Non-GM cotton in the west of greenhouse was set up as the control group and non-GM cotton in the east of greenhouse as the treatment group. Bars indicate sampling points.

The GM cotton was planted in a 4 m × 8 m square in the middle of a 60 m × 8 m square area of non-GM cotton according to the scheme map presented in Fig. 3. Rows were spaced 0.8 m apart and plants within rows were spaced 0.5 m apart. Non-GM cotton in the west of greenhouse was set up as the control group and non-GM cotton in the east of greenhouse as the treatment group. Insect proof net (9 holes/cm^2^) was used to prevent pollinators moving into the control groups, and did not influence the pollen transfer. All plants were monitored at least twice weekly for florescence emergence and anthesis from early July through late August to ensure the flowering synchrony between GM and non-GM cotton^37^. Pollinators were released in three greenhouses in early August (25–28 °C, full-bloom stage for all plants). At harvest, seeds from pollen receptors were obtained by hand from lower, middle, and upper flowers at the distance of 0.8, 1.6, 3.2, 6.4, 12.8, 19.2, 25.6 and 36 m for the treatment group and 0.8, 1.6, 3.2, 6.4, 12.8 and 19.2 m for the control group from the donor plot when mature but before excessive shattering^19^. No significant differences in PGF were noted for flower positions on the pollen receptors^16^. Thus, seeds from lower, middle, and upper flowers were set up as three replications. Cotton was ginned separately, acid delinted, mixed and sub-sampled for the following detection.

### Hybrid confirmation

Seeds of the F_1_ generation, from the three greenhouses, were planted in flats containing moist potting mix composed of 75% vermiculite and 25% turf (by volume) in a greenhouse with a temperature of 22–35 °C and a 14 h photoperiod. Control flats were also seeded and evaluated, including the seeds of Shiyuan321 and Hai7124. Seedlings were allowed to grow to the 3-leaf stage, and one part of each seedling was used for DNA extraction and polymerase chain reaction (PCR) amplification, and the seedling grew continually. For each distance point, we screened 60 seedlings from each pollen receptor for hybrid confirmation.

The presence of transgenic DNA in putative hybrids was verified by PCR amplification of the fragment sequence of *Cry I Ac*. DNA was extracted from the leaf of a seedling using the cetyltrimethyl ammonium bromide (CTAB) protocol^56^ with the addition of 20 g/L polyvinylpyrrolidone (PVP) in the extraction buffer. A 1 μL sample of DNA was used to perform spectroscopic quantitation using a NanoDrop 2000 spectrophotometer (Thermo Fisher, USA). Primers of 5′-GAAGGATTGAGCAATCTCTAC-3′ and 5′-CAATCAGCCTAGTAAGGTCGT-3′ were used to amplify the *Cry I Ac* with 80 ng of DNA^20^. The PCR condition was: 95 °C for 4 min, followed by 30 cycles at 94 °C for 60 s, 56 °C for 60 s, 72 °C for 90 s, and a final extension step at 72 °C for 5 min^20^. Routine screening for PCR assay was performed by agarose gel electrophoresis (10 g agarose/L) with GM cotton as positive control and non-GM Shiyuan321 as negative control.


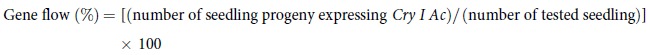


Total protein was extracted from 0.1 g of a leaf of seedling progeny expressing *Cry I Ac*, and was applied for Bt protein detection using Bt-Cry 1 Ab/Ac detection kits (Beijing Yintu Biological Technology Co., Beijing, China) in accordance with a previously described method^20^ (Figure S3). The appearance of a band indicated it was positive.


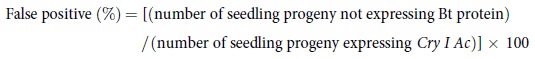


### Biological assay for detection of hybrid

The confirmed hybrid expressing Bt protein (cross-pollination with Shiyuan321), non-GM cultivar (Shiyuan321), and GM cotton were sprayed with a 0.25% (v/v) glyphosate solution at the 4-leaf stage, respectively. The height of a seedling that survived was measured at 0, 7, 14, 28 days after glyphosate application^57^, and the survival rate was recorded at 42 d after glyphosate application. Each treatment contained 50 seedlings, which was repeated 3 times.

*Helicoverpa armigera* larvae were used to determine the insect resistance of confirmed hybrids. *H. armigera* collected from a cotton field were reared in the IPM lab (China Agricultural University, 40°02′N, 116°28<mml:mspace width=”0.25em” depth=”0000”/>E) at 27 ± 1 °C, 75 ± 10% relative humidity (RH) and a 14: 10 light: dark photoperiod. This species has been cultured for more than 10 yr without exposure to Bt or other insecticides^58^,^59^. Larvae were reared on a synthetic diet^60^ and adults were supplied with a 10% honey solution. In the bioassay, larvae were reared on the artificial diet until the third instar. Then they were transferred into glass tubes (1 cm × 6 cm) containing leaves at the 5-leaf stage from the confirmed hybrid (cross-pollination with Shiyuan321), non-GM cotton (Shiyuan321 as control) or GM cotton according to the method described by Liu *et al.*^58^. Six d later, the weight and mortality of *H. armigera* larvae were recorded^57^. Each treatment contained 60 larvae, which was repeated 3 times.

### Data analysis

Descriptive statistics were given as the mean values and standard errors of the mean. The data were analyzed by Tukey’s multiple comparison test at the *P* = 0.05 level of significance. Statistics were performed with SPSS 16.0 software (IBM, Armonk, NY). Regression analyses were performed using SigmaPlot 10.0 software (Systat Software Inc., San Jose). The correlation coefficient (R^2^) between PGF and distance was obtained from SigmaPlot 10.0 software.

## Additional Information

**How to cite this article**: Yan, S. *et al.* Pollen-mediated gene flow from transgenic cotton under greenhouse conditions is dependent on different pollinators. *Sci. Rep.* **5**, 15917; doi: 10.1038/srep15917 (2015).

## Supplementary Material

Supplementary Information

## Figures and Tables

**Table 1:** Pollen-mediated gene flow frequency with different pollinators from GM cotton to Shiyuan321 and Hai7124 during 2012 and 2013 (%).

Distance(m)	Shiyuan321			Hai7124		
	*B. ignitus*	*A. mellifer*	*P. rapae*	*B. ignitus*	*A. mellifer*	*P. rapae*
2012						
0.8	26.67 ± 6.67	20.00 ± 2.89	13.33 ± 3.33	20.00 ± 5.00	3.33 ± 3.33	1.67 ± 1.67
1.6	30.00 ± 5.77	18.33 ± 3.33	6.67 ± 1.67	13.33 ± 3.33	6.67 ± 4.41	0.00 ± 0.00
3.2	21.67 ± 4.41	11.67 ± 4.41	3.33 ± 3.33	21.67 ± 6.01	5.00 ± 5.00	1.67 ± 1.67
6.4	28.33 ± 6.01	16.67 ± 4.41	5.00 ± 2.89	15.00 ± 2.89	0.00 ± 0.00	1.67 ± 1.67
12.8	5.00 ± 2.89	3.33 ± 3.33	3.33 ± 1.67	1.67 ± 1.67	0.00 ± 0.00	3.33 ± 3.33
19.2	0.00 ± 0.00	1.67 ± 1.67	0.00 ± 0.00	5.00 ± 2.89	1.67 ± 1.67	1.67 ± 1.67
25.6	3.33 ± 1.67	0.00 ± 0.00	1.67 ± 1.67	0.00 ± 0.00	1.67 ± 1.67	0.00 ± 0.00
36	0.00 ± 0.00	0.00 ± 0.00	0.00 ± 0.00	0.00 ± 0.00	0.00 ± 0.00	1.67 ± 1.67
Average	14.38 ± 2.91	8.96 ± 1.90	4.17 ± 1.07	9.58 ± 2.02	2.29 ± 0.95	1.46 ± 0.56
2013						
0.8	18.33 ± 4.41	8.33 ± 3.33	5.00 ± 2.89	11.67 ± 4.41	1.67 ± 1.67	1.67 ± 1.67
1.6	21.67 ± 4.41	16.67 ± 4.41	11.67 ± 4.41	10.00 ± 2.89	5.00 ± 5.00	1.67 ± 1.67
3.2	15.00 ± 2.89	11.67 ± 3.33	6.67 ± 3.33	15.00 ± 2.89	5.00 ± 2.89	0.00 ± 0.00
6.4	5.00 ± 2.89	11.67 ± 3.33	0.00 ± 0.00	11.67 ± 1.67	5.00 ± 2.89	1.67 ± 1.67
12.8	11.67 ± 4.41	8.33 ± 1.67	1.67 ± 1.67	6.67 ± 4.41	0.00 ± 0.00	0.00 ± 0.00
19.2	11.67 ± 3.33	6.67 ± 3.33	1.67 ± 1.67	5.00 ± 2.89	1.67 ± 1.67	1.67 ± 1.67
25.6	1.67 ± 1.67	0.00 ± 0.00	5.00 ± 5.00	0.00 ± 0.00	1.67 ± 1.67	1.67 ± 1.67
36	8.33 ± 1.67	3.33 ± 1.67	1.67 ± 1.67	1.67 ± 1.67	0.00 ± 0.00	0.00 ± 0.00
Average	11.67 ± 1.64	8.33 ± 1.34	4.17 ± 1.15	7.71 ± 1.35	2.50 ± 0.85	1.04 ± 0.42

Means ± SE. Seeds from lower, middle, and upper flowers were set up as three replications.

**Table 2:** Glyphosate resistance detection of confirmed hybrid, Shiyuan321, and GM cotton.

Concentration (%)		Height of cotton (cm)				survival rate (%)
		0 d	7 d	14 d	2814 d	
GM cotton	0.25	26.47 ± 0.83 A	35.47 ± 0.64 A	44.80 ± 1.10 A	54.87 ± 1.36 AB	100.00 ± 0.00 A
Confirmed hybrid	0.25	26.83 ± 0.55 A	31.77 ± 0.59 B	39.27 ± 0.90 B	50.97 ± 1.39 B	82.78 ± 2.42 B
Shiyuan321	0.25	26.70 ± 1.13 A	28.13 ± 0.90 C	30.27 ± 1.25 C	31.03 ± 1.84 C	0.00 ± 0.00 C
GM cotton	0	26.37 ± 0.88 A	37.30 ± 0.55 A	46.03 ± 0.87 A	58.30 ± 1.21 A	100.00 ± 0.00 A
Confirmed hybrid	0	26.63 ± 0.73 A	36.60 ± 0.64 A	45.37 ± 0.79 A	56.17 ± 0.79 AB	100.00 ± 0.00 A
Shiyuan321	0	26.40 ± 0.60 A	36.43 ± 0.39 A	44.87 ± 0.65 A	56.40 ± 0.62 AB	100.00 ± 0.00 A
*F*	−	0.053	31.909	42.022	65.275	1638.000
*P*	−	0.998	<0.001	<0.001	<0.001	<0.001

Means ± SE followed by different letters are significantly different (Tukey’s multiple comparison test, *P* < 0.05). Each treatment contained 50 seedlings, which was repeated 3 times.
