# Supplementary material for: Pollen-mediated gene flow from transgenic cotton under greenhouse conditions is dependent on different pollinators
Source: Sci Rep. 2015 Nov 3;5:15917. doi: 10.1038/srep15917 (PMC4630633; doi:10.1038/srep15917)

**Pollen-mediated gene flow from transgenic cotton under greenhouseconditions is dependent on different pollinators**

**Shuo Yan^1,2^, Jialin Zhu^1,3^, Weilong Zhu^1^, Zhen Li^1^, Anthony M. Shelton^4^, Junyu Luo^5^,Jinjie Cui^5^, Qingwen Zhang^1^* and Xiaoxia Liu^1^***

1 Department of Entomology, China Agricultural University, Beijing, P.R. China, 100193

2 National Agricultural Technology Extension and Service Center, Beijing, P.R. China,100125

3 Beijing Entry-Exit Inspection and Quarantine Bureau, Beijing, P.R. China, 100026

4 Department of Entomology, Cornell University/New York State Agricultural ExperimentStation, Geneva, NY, USA, 14456

5 State Key Laboratory of Cotton Biology, Institute of Cotton Research, Chinese Academyof Agricultural Sciences, Anyang , Henan, P.R. China, 455000

*Corresponding author: Xiaoxia Liu (liuxiaoxia611@cau.edu.cn) and Qingwen Zhang(zhangqingwen@263.net)

**Additional Information**

**Figure S1 The measurement of wind velocity in varied treatments.**


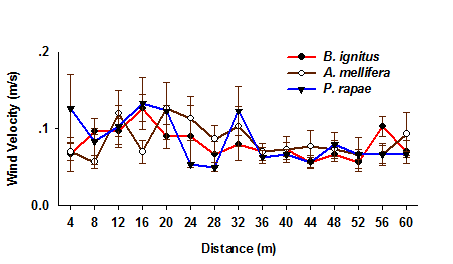


**Figure S2 PCR analysis of DNA from tested seedlings.** CK-: the negative control (Shiyuan321); CK+: the positive control (GM cotton); 1-19: tested seedlings.


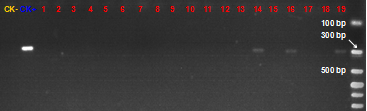


**Figure S3 Detection of Bt protein using Bt-Cry 1 Ab/Ac detection kits.** A: true scene of Bt protein detection; B: schematic diagram of Bt protein detection.


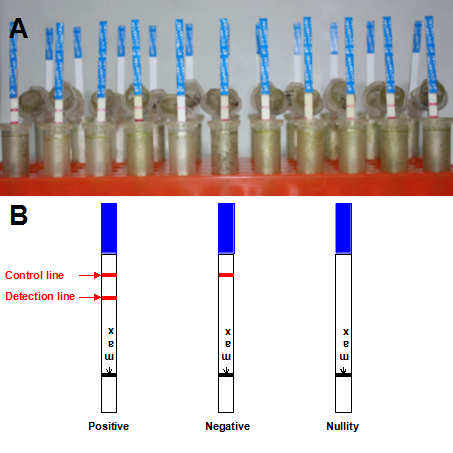

Supplement: Supplementary Information [file srep15917-s1.doc]
